# (*E*)-1-{4-[Bis(4-bromo­phen­yl)meth­yl]piperazin-1-yl}-3-(4-bromo­phen­yl)prop-2-en-1-one

**DOI:** 10.1107/S1600536811048380

**Published:** 2011-11-19

**Authors:** Yan Zhong, XiaoPing Zhang, Bin Wu

**Affiliations:** a School of Chemistry and Chemical Engineering, Southeast University, Sipailou No. 2 Nanjing, Nanjing 210096, People’s Republic of China; b Centre of Laboratory Animals, Nanjing Medical University, Hanzhong Road No. 140 Nanjing, Nanjing 210029, People’s Republic of China; c School of Pharmacy, Nanjing Medical University, Hanzhong Road No. 140 Nanjing, Nanjing 210029, People’s Republic of China

## Abstract

In the title mol­ecule, C_26_H_23_Br_3_N_2_O, the piperazine ring adopts a chair conformation and the C=C double bond has an *E* configuration. In the crystal, mol­ecules are linked through weak inter­molecular C—H⋯O hydrogen bonds.

## Related literature

For pharmacological properties of cinnamic acid derivatives, see: Shi *et al.* (2005 ▶); Qian *et al.* (2010 ▶). For the synthesis of the title compound, see: Wu *et al.* (2008 ▶). For a related structure, see: Teng *et al.* (2011 ▶). For puckering parameters, see: Cremer & Pople (1975 ▶).
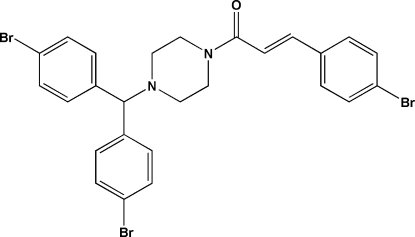


## Experimental

### 

#### Crystal data

C_26_H_23_Br_3_N_2_O
*M* *_r_* = 619.19
Monoclinic, 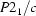

*a* = 9.956 (2) Å
*b* = 11.624 (2) Å
*c* = 21.310 (4) Å
β = 101.45 (3)°
*V* = 2417.1 (8) Å^3^
*Z* = 4
Mo *K*α radiation
μ = 5.03 mm^−1^
*T* = 293 K
0.20 × 0.10 × 0.10 mm

#### Data collection

Enraf–Nonius CAD-4 diffractometer
Absorption correction: ψ scan (North *et al.*, 1968 ▶) *T*_min_ = 0.433, *T*_max_ = 0.633
4701 measured reflections
4432 independent reflections
2081 reflections with *I* > 2σ(*I*)
*R*_int_ = 0.098
3 standard reflections every 200 reflections  intensity decay: 1%

#### Refinement

*R*[*F* ^2^ > 2σ(*F* ^2^)] = 0.069
*wR*(*F* ^2^) = 0.082
*S* = 1.01
4432 reflections
289 parameters
2 restraints
H-atom parameters constrained
Δρ_max_ = 0.35 e Å^−3^
Δρ_min_ = −0.42 e Å^−3^

### 

Data collection: *CAD-4 EXPRESS* (Enraf–Nonius, 1994 ▶); cell refinement: *CAD-4 EXPRESS*; data reduction: *XCAD4* (Harms & Wocadlo, 1995 ▶); program(s) used to solve structure: *SHELXS97* (Sheldrick, 2008 ▶); program(s) used to refine structure: *SHELXL97* (Sheldrick, 2008 ▶); molecular graphics: *SHELXTL* (Sheldrick, 2008 ▶); software used to prepare material for publication: *SHELXL97*.

## Supplementary Material

Crystal structure: contains datablock(s) I, global. DOI: 10.1107/S1600536811048380/pv2478sup1.cif

Structure factors: contains datablock(s) I. DOI: 10.1107/S1600536811048380/pv2478Isup2.hkl

Supplementary material file. DOI: 10.1107/S1600536811048380/pv2478Isup3.cml

Additional supplementary materials:  crystallographic information; 3D view; checkCIF report

## Figures and Tables

**Table 1:** Hydrogen-bond geometry (Å, °)

*D*—H⋯*A*	*D*—H	H⋯*A*	*D*⋯*A*	*D*—H⋯*A*
C20—H20*A*⋯O^i^	0.93	2.60	3.480 (7)	159

Symmetry code: (i) 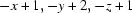.

**Fig. 1.**
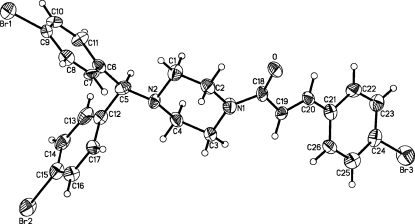
The molecular structure and numbering scheme of the title compound; displacement ellipsoids are drawn at the 70% probability level.

**Fig. 2.**
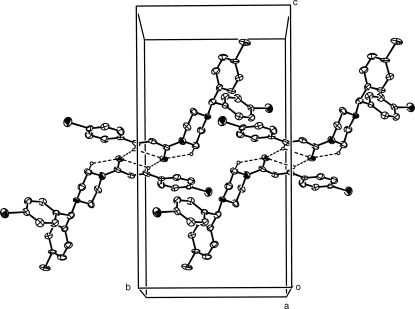
A view of the unit cell of the title compound showing intermolecular and intramolecular hydrogen bonds.

## supplementary crystallographic information

### Comment

Recently, many compounds containing a cinnamoyl moiety have drawn much attention owing to their significant pharmacological properties such as antimicrobial, anticancer and neuroprotective activities (Shi *et al.*, 2005; Qian *et al.*, 2010). As a part of our ongoing study of the substituent effect on the stuctures of cinnamide derivatives, we report herein the crystal structure of the title compound.

The title compound (Fig. 1) exhibits an E configulation with respect to the C19═C20 ethene bond [1.320 (7) Å] with a torsion angle C18—C19—C20—C21 = -177.4 (6)°. The piperazine ring adopts a chair conformation with puchering parameters (Cremer & Pople, 1975) *Q* = 0.542 (6)Å, θ = 4.6 (6)° and φ = 157 (9)°. In the crystal, molecules are linked by intermolecular C—H···O interactions (Tab. 1, Fig. 2).

### Experimental

The synthesis follows the method of Wu *et al.* (2008). The title compound was prepared by stirring a mixture of (*E*)-3-(4-bromophenyl)acrylic acid (0.908 g, 4 mmol), dimethyl sulfoxide (2 ml) and dichloromethane (30 ml) for 6 h at room temperature. The solvent was removed under reduced pressure. The residue was dissolved in acetone (15 ml) and reacted with 1-(bis(4-bromophenyl)methyl) piperazine (2.461 g, 6 mmol) in the presence of triethylamine (5 ml) for 12 h at room temperature. The resultant mixture was cooled. The title compound thus obtained was filtered and recrystallized from ethanol. The pale-yellow single crystals of the title compound used in *X*-ray diffraction studies were grown from a mixture of ethanol and chloroform (2:1) by slow evaporation at room temperature.

### Refinement

The hydrogen atoms were positioned geometrically with C—H distances 0.93, 0.97 and 0.98 Å for aryl, methyne and methylene type H-atoms, respectively, and refined as riding on their parent atoms with *U*_iso_(H) = 1.2 *U*_eq_ of the carrier atom.

### Crystal data

**Table:**

C_26_H_23_Br_3_N_2_O	*F*(000) = 1224
*M**_r_* = 619.19	*D*_x_ = 1.702 Mg m^−^^3^
Monoclinic, *P*2_1_/*c*	Mo *K*α radiation, λ = 0.71073 Å
Hall symbol: -P 2ybc	Cell parameters from 25 reflections
*a* = 9.956 (2) Å	θ = 10–13°
*b* = 11.624 (2) Å	µ = 5.03 mm^−^^1^
*c* = 21.310 (4) Å	*T* = 293 K
β = 101.45 (3)°	Block, pale-yellow
*V* = 2417.1 (8) Å^3^	0.20 × 0.10 × 0.10 mm
*Z* = 4	

### Data collection

**Table:**

Enraf–Nonius CAD-4 diffractometer	2081 reflections with *I* > 2σ(*I*)
Radiation source: fine-focus sealed tube	*R*_int_ = 0.098
graphite	θ_max_ = 25.4°, θ_min_ = 2.0°
ω/2θ scans	*h* = 0→12
Absorption correction: ψ scan (North *et al.*, 1968)	*k* = 0→14
*T*_min_ = 0.433, *T*_max_ = 0.633	*l* = −25→25
4701 measured reflections	3 standard reflections every 200 reflections
4432 independent reflections	intensity decay: 1%

### Refinement

**Table:**

Refinement on *F*^2^	Primary atom site location: structure-invariant direct methods
Least-squares matrix: full	Secondary atom site location: difference Fourier map
*R*[*F*^2^ > 2σ(*F*^2^)] = 0.069	Hydrogen site location: inferred from neighbouring sites
*wR*(*F*^2^) = 0.082	H-atom parameters constrained
*S* = 1.01	*w* = 1/[σ^2^(*F*_o_^2^) + (0.0185*P*)^2^] where *P* = (*F*_o_^2^ + 2*F*_c_^2^)/3
4432 reflections	(Δ/σ)_max_ = 0.001
289 parameters	Δρ_max_ = 0.35 e Å^−^^3^
2 restraints	Δρ_min_ = −0.42 e Å^−^^3^

### Special details

**Table:**

Geometry. All e.s.d.'s (except the e.s.d. in the dihedral angle between two l.s. planes) are estimated using the full covariance matrix. The cell e.s.d.'s are taken into account individually in the estimation of e.s.d.'s in distances, angles and torsion angles; correlations between e.s.d.'s in cell parameters are only used when they are defined by crystal symmetry. An approximate (isotropic) treatment of cell e.s.d.'s is used for estimating e.s.d.'s involving l.s. planes.
Refinement. Refinement of *F*^2^ against ALL reflections. The weighted *R*-factor *wR* and goodness of fit *S* are based on *F*^2^, conventional *R*-factors *R* are based on *F*, with *F* set to zero for negative *F*^2^. The threshold expression of *F*^2^ > σ(*F*^2^) is used only for calculating *R*-factors(gt) *etc*. and is not relevant to the choice of reflections for refinement. *R*-factors based on *F*^2^ are statistically about twice as large as those based on *F*, and *R*- factors based on ALL data will be even larger.

### Fractional atomic coordinates and isotropic or equivalent isotropic displacement parameters (Å^2^)

**Table:**

	*x*	*y*	*z*	*U*_iso_*/*U*_eq_	
O	0.4281 (4)	0.8456 (3)	0.49348 (19)	0.0564 (13)	
Br1	0.93912 (8)	0.12182 (7)	0.72681 (4)	0.0763 (3)	
N1	0.3393 (5)	0.7165 (5)	0.5513 (3)	0.0582 (15)	
C1	0.5229 (6)	0.5927 (5)	0.6057 (3)	0.059 (2)	
H1A	0.5859	0.5328	0.5984	0.071*	
H1B	0.5767	0.6573	0.6257	0.071*	
Br2	0.16720 (8)	0.29610 (8)	0.88918 (3)	0.0834 (3)	
N2	0.4370 (5)	0.5493 (4)	0.6490 (2)	0.0443 (13)	
C2	0.4386 (6)	0.6302 (5)	0.5429 (3)	0.0562 (18)	
H2A	0.4984	0.6611	0.5162	0.067*	
H2B	0.3913	0.5641	0.5211	0.067*	
Br3	−0.05208 (8)	1.44635 (7)	0.58711 (4)	0.0847 (3)	
C3	0.2520 (6)	0.6816 (5)	0.5959 (3)	0.0557 (19)	
H3A	0.1902	0.6210	0.5768	0.067*	
H3B	0.1975	0.7465	0.6048	0.067*	
C4	0.3417 (6)	0.6387 (5)	0.6579 (3)	0.0549 (18)	
H4A	0.3929	0.7032	0.6795	0.066*	
H4B	0.2829	0.6098	0.6856	0.066*	
C5	0.5188 (6)	0.5151 (5)	0.7109 (3)	0.0510 (18)	
H5A	0.5632	0.5843	0.7316	0.061*	
C6	0.6277 (6)	0.4288 (5)	0.7081 (3)	0.0457 (16)	
C7	0.6099 (5)	0.3340 (5)	0.6655 (3)	0.0483 (17)	
H7A	0.5309	0.3315	0.6337	0.058*	
C8	0.7002 (7)	0.2476 (6)	0.6681 (3)	0.0563 (19)	
H8A	0.6839	0.1876	0.6387	0.068*	
C9	0.8187 (6)	0.2489 (5)	0.7156 (3)	0.0473 (17)	
C10	0.8421 (6)	0.3419 (6)	0.7559 (3)	0.059 (2)	
H10A	0.9232	0.3460	0.7862	0.071*	
C11	0.7479 (6)	0.4292 (6)	0.7522 (3)	0.0563 (19)	
H11A	0.7665	0.4905	0.7806	0.068*	
C12	0.4301 (6)	0.4650 (5)	0.7545 (3)	0.0461 (16)	
C13	0.4486 (6)	0.4907 (6)	0.8175 (3)	0.062 (2)	
H13A	0.5143	0.5455	0.8340	0.075*	
C14	0.3732 (6)	0.4388 (6)	0.8595 (3)	0.062 (2)	
H14A	0.3908	0.4564	0.9029	0.075*	
C15	0.2737 (7)	0.3620 (6)	0.8339 (3)	0.0552 (19)	
C16	0.2504 (6)	0.3338 (5)	0.7715 (3)	0.0543 (18)	
H16A	0.1823	0.2810	0.7550	0.065*	
C17	0.3289 (6)	0.3840 (5)	0.7315 (3)	0.0494 (17)	
H17A	0.3135	0.3631	0.6886	0.059*	
C18	0.3496 (7)	0.8259 (6)	0.5292 (3)	0.0500 (17)	
C19	0.2533 (6)	0.9125 (5)	0.5439 (3)	0.0452 (17)	
H19A	0.1741	0.8893	0.5573	0.054*	
C20	0.2785 (6)	1.0231 (6)	0.5384 (3)	0.0491 (17)	
H20A	0.3574	1.0400	0.5231	0.059*	
C21	0.2005 (6)	1.1230 (6)	0.5527 (3)	0.0466 (16)	
C22	0.2556 (6)	1.2308 (6)	0.5522 (3)	0.0543 (18)	
H22A	0.3449	1.2390	0.5460	0.065*	
C23	0.1802 (7)	1.3288 (6)	0.5610 (3)	0.061 (2)	
H23A	0.2167	1.4020	0.5588	0.074*	
C24	0.0504 (7)	1.3141 (6)	0.5729 (3)	0.0522 (18)	
C25	−0.0090 (7)	1.2091 (6)	0.5737 (3)	0.0583 (19)	
H25A	−0.0983	1.2011	0.5800	0.070*	
C26	0.0699 (6)	1.1140 (6)	0.5648 (3)	0.0586 (19)	
H26A	0.0329	1.0411	0.5671	0.070*	

### Atomic displacement parameters (Å^2^)

**Table:**

	*U*^11^	*U*^22^	*U*^33^	*U*^12^	*U*^13^	*U*^23^
O	0.067 (3)	0.065 (3)	0.043 (3)	−0.005 (2)	0.024 (2)	0.008 (2)
Br1	0.0670 (5)	0.0714 (5)	0.0907 (7)	0.0136 (4)	0.0157 (4)	0.0143 (5)
N1	0.065 (4)	0.053 (4)	0.060 (4)	0.011 (3)	0.019 (3)	0.011 (3)
C1	0.061 (5)	0.067 (5)	0.052 (5)	0.016 (4)	0.017 (4)	0.003 (4)
Br2	0.0751 (6)	0.1359 (8)	0.0414 (5)	0.0163 (6)	0.0169 (4)	0.0266 (5)
N2	0.051 (3)	0.052 (3)	0.028 (3)	0.008 (3)	0.004 (2)	0.007 (3)
C2	0.063 (4)	0.061 (4)	0.048 (5)	−0.004 (4)	0.019 (4)	0.007 (4)
Br3	0.0988 (7)	0.0678 (5)	0.0948 (7)	0.0127 (5)	0.0369 (5)	−0.0054 (5)
C3	0.049 (4)	0.060 (5)	0.059 (5)	0.003 (4)	0.014 (4)	0.021 (4)
C4	0.059 (4)	0.051 (4)	0.063 (5)	0.008 (4)	0.030 (4)	0.016 (4)
C5	0.044 (4)	0.059 (4)	0.049 (5)	0.001 (3)	0.004 (3)	−0.012 (4)
C6	0.054 (4)	0.048 (4)	0.035 (4)	−0.012 (4)	0.008 (3)	0.002 (4)
C7	0.025 (3)	0.065 (5)	0.050 (4)	−0.011 (3)	−0.005 (3)	−0.001 (4)
C8	0.063 (5)	0.054 (5)	0.052 (5)	−0.005 (4)	0.012 (4)	−0.007 (4)
C9	0.044 (4)	0.043 (4)	0.055 (5)	0.001 (3)	0.011 (4)	0.014 (4)
C10	0.035 (4)	0.070 (5)	0.073 (6)	−0.010 (4)	0.015 (4)	0.007 (4)
C11	0.043 (4)	0.070 (5)	0.053 (5)	0.008 (4)	0.002 (3)	−0.011 (4)
C12	0.048 (4)	0.046 (4)	0.042 (5)	0.004 (3)	0.003 (3)	−0.007 (3)
C13	0.062 (5)	0.075 (5)	0.042 (5)	0.017 (4)	−0.009 (4)	−0.008 (4)
C14	0.055 (5)	0.096 (6)	0.033 (5)	0.017 (4)	−0.001 (4)	0.006 (4)
C15	0.065 (5)	0.083 (5)	0.022 (4)	0.016 (4)	0.019 (3)	0.007 (4)
C16	0.057 (4)	0.064 (5)	0.042 (4)	0.000 (4)	0.010 (3)	−0.009 (4)
C17	0.052 (4)	0.071 (4)	0.026 (4)	−0.003 (4)	0.011 (3)	0.008 (4)
C18	0.060 (4)	0.056 (4)	0.034 (4)	0.004 (4)	0.009 (3)	0.017 (4)
C19	0.040 (3)	0.063 (5)	0.032 (4)	0.005 (4)	0.005 (3)	0.013 (3)
C20	0.050 (4)	0.062 (5)	0.033 (4)	−0.013 (4)	0.000 (3)	0.016 (4)
C21	0.065 (4)	0.056 (4)	0.019 (4)	0.000 (4)	0.009 (3)	−0.001 (3)
C22	0.049 (4)	0.068 (5)	0.039 (4)	−0.013 (4)	−0.007 (3)	−0.006 (4)
C23	0.068 (5)	0.064 (5)	0.051 (5)	0.009 (4)	0.009 (4)	0.005 (4)
C24	0.074 (5)	0.050 (4)	0.026 (4)	0.005 (4)	−0.007 (3)	−0.011 (3)
C25	0.062 (5)	0.069 (5)	0.045 (4)	0.002 (4)	0.012 (3)	−0.008 (4)
C26	0.053 (4)	0.057 (5)	0.071 (5)	−0.003 (4)	0.022 (4)	0.015 (4)

### Geometric parameters (Å, °)

**Table:**

O—C18	1.216 (6)	C9—C10	1.372 (8)
Br1—C9	1.887 (6)	C10—C11	1.373 (7)
N1—C18	1.368 (7)	C10—H10A	0.9300
N1—C2	1.444 (7)	C11—H11A	0.9300
N1—C3	1.466 (6)	C12—C13	1.351 (7)
C1—N2	1.467 (6)	C12—C17	1.394 (7)
C1—C2	1.498 (7)	C13—C14	1.413 (6)
C1—H1A	0.9700	C13—H13A	0.9300
C1—H1B	0.9700	C14—C15	1.365 (8)
Br2—C15	1.897 (6)	C14—H14A	0.9300
N2—C4	1.445 (6)	C15—C16	1.344 (8)
N2—C5	1.460 (7)	C16—C17	1.393 (7)
C2—H2A	0.9700	C16—H16A	0.9300
C2—H2B	0.9700	C17—H17A	0.9300
Br3—C24	1.902 (6)	C18—C19	1.466 (7)
C3—C4	1.524 (7)	C19—C20	1.320 (7)
C3—H3A	0.9700	C19—H19A	0.9300
C3—H3B	0.9700	C20—C21	1.462 (8)
C4—H4A	0.9700	C20—H20A	0.9300
C4—H4B	0.9700	C21—C22	1.368 (8)
C5—C6	1.488 (7)	C21—C26	1.379 (7)
C5—C12	1.520 (7)	C22—C23	1.396 (7)
C5—H5A	0.9800	C22—H22A	0.9300
C6—C11	1.367 (7)	C23—C24	1.377 (7)
C6—C7	1.417 (6)	C23—H23A	0.9300
C7—C8	1.341 (7)	C24—C25	1.358 (7)
C7—H7A	0.9300	C25—C26	1.390 (7)
C8—C9	1.394 (8)	C25—H25A	0.9300
C8—H8A	0.9300	C26—H26A	0.9300
			
C18—N1—C2	120.2 (5)	C11—C10—H10A	119.4
C18—N1—C3	125.1 (6)	C6—C11—C10	122.1 (7)
C2—N1—C3	113.4 (5)	C6—C11—H11A	119.0
N2—C1—C2	111.6 (5)	C10—C11—H11A	119.0
N2—C1—H1A	109.3	C13—C12—C17	116.4 (6)
C2—C1—H1A	109.3	C13—C12—C5	122.8 (6)
N2—C1—H1B	109.3	C17—C12—C5	120.7 (6)
C2—C1—H1B	109.3	C12—C13—C14	123.4 (7)
H1A—C1—H1B	108.0	C12—C13—H13A	118.3
C4—N2—C5	110.0 (5)	C14—C13—H13A	118.3
C4—N2—C1	108.3 (5)	C15—C14—C13	117.4 (6)
C5—N2—C1	111.8 (5)	C15—C14—H14A	121.3
N1—C2—C1	111.5 (5)	C13—C14—H14A	121.3
N1—C2—H2A	109.3	C16—C15—C14	121.6 (6)
C1—C2—H2A	109.3	C16—C15—Br2	120.6 (6)
N1—C2—H2B	109.3	C14—C15—Br2	117.8 (5)
C1—C2—H2B	109.3	C15—C16—C17	119.7 (6)
H2A—C2—H2B	108.0	C15—C16—H16A	120.2
N1—C3—C4	109.3 (5)	C17—C16—H16A	120.2
N1—C3—H3A	109.8	C16—C17—C12	121.4 (6)
C4—C3—H3A	109.8	C16—C17—H17A	119.3
N1—C3—H3B	109.8	C12—C17—H17A	119.3
C4—C3—H3B	109.8	O—C18—N1	119.5 (6)
H3A—C3—H3B	108.3	O—C18—C19	122.3 (6)
N2—C4—C3	114.2 (5)	N1—C18—C19	117.9 (6)
N2—C4—H4A	108.7	C20—C19—C18	120.4 (6)
C3—C4—H4A	108.7	C20—C19—H19A	119.8
N2—C4—H4B	108.7	C18—C19—H19A	119.8
C3—C4—H4B	108.7	C19—C20—C21	129.6 (6)
H4A—C4—H4B	107.6	C19—C20—H20A	115.2
N2—C5—C6	115.4 (5)	C21—C20—H20A	115.2
N2—C5—C12	111.6 (5)	C22—C21—C26	117.7 (6)
C6—C5—C12	106.4 (5)	C22—C21—C20	119.7 (6)
N2—C5—H5A	107.7	C26—C21—C20	122.5 (6)
C6—C5—H5A	107.7	C21—C22—C23	121.3 (6)
C12—C5—H5A	107.7	C21—C22—H22A	119.4
C11—C6—C7	115.2 (6)	C23—C22—H22A	119.4
C11—C6—C5	121.1 (6)	C24—C23—C22	118.2 (6)
C7—C6—C5	123.4 (6)	C24—C23—H23A	120.9
C8—C7—C6	123.9 (6)	C22—C23—H23A	120.9
C8—C7—H7A	118.1	C25—C24—C23	122.7 (6)
C6—C7—H7A	118.1	C25—C24—Br3	118.4 (6)
C7—C8—C9	119.1 (6)	C23—C24—Br3	118.8 (5)
C7—C8—H8A	120.4	C24—C25—C26	117.0 (6)
C9—C8—H8A	120.4	C24—C25—H25A	121.5
C10—C9—C8	118.5 (6)	C26—C25—H25A	121.5
C10—C9—Br1	120.9 (5)	C21—C26—C25	123.0 (6)
C8—C9—Br1	120.6 (5)	C21—C26—H26A	118.5
C9—C10—C11	121.2 (6)	C25—C26—H26A	118.5
C9—C10—H10A	119.4		
			
C2—C1—N2—C4	−57.4 (7)	C6—C5—C12—C17	−80.8 (7)
C2—C1—N2—C5	−178.7 (5)	C17—C12—C13—C14	1.1 (10)
C18—N1—C2—C1	114.1 (6)	C5—C12—C13—C14	−175.5 (6)
C3—N1—C2—C1	−53.7 (7)	C12—C13—C14—C15	−2.4 (10)
N2—C1—C2—N1	56.8 (7)	C13—C14—C15—C16	1.9 (10)
C18—N1—C3—C4	−116.3 (7)	C13—C14—C15—Br2	−177.7 (5)
C2—N1—C3—C4	50.7 (7)	C14—C15—C16—C17	−0.2 (10)
C5—N2—C4—C3	179.4 (5)	Br2—C15—C16—C17	179.4 (5)
C1—N2—C4—C3	57.0 (7)	C15—C16—C17—C12	−1.2 (10)
N1—C3—C4—N2	−53.7 (7)	C13—C12—C17—C16	0.7 (9)
C4—N2—C5—C6	−176.0 (5)	C5—C12—C17—C16	177.4 (6)
C1—N2—C5—C6	−55.7 (7)	C2—N1—C18—O	11.9 (10)
C4—N2—C5—C12	62.3 (6)	C3—N1—C18—O	178.2 (6)
C1—N2—C5—C12	−177.3 (5)	C2—N1—C18—C19	−174.8 (5)
N2—C5—C6—C11	147.7 (6)	C3—N1—C18—C19	−8.5 (10)
C12—C5—C6—C11	−87.9 (7)	O—C18—C19—C20	−23.9 (10)
N2—C5—C6—C7	−39.8 (8)	N1—C18—C19—C20	162.9 (6)
C12—C5—C6—C7	84.6 (7)	C18—C19—C20—C21	−177.4 (6)
C11—C6—C7—C8	2.1 (9)	C19—C20—C21—C22	170.6 (6)
C5—C6—C7—C8	−170.8 (6)	C19—C20—C21—C26	−11.8 (10)
C6—C7—C8—C9	0.6 (10)	C26—C21—C22—C23	−2.6 (9)
C7—C8—C9—C10	−3.4 (9)	C20—C21—C22—C23	175.2 (6)
C7—C8—C9—Br1	173.4 (4)	C21—C22—C23—C24	2.7 (9)
C8—C9—C10—C11	3.6 (9)	C22—C23—C24—C25	−2.9 (10)
Br1—C9—C10—C11	−173.2 (5)	C22—C23—C24—Br3	178.4 (5)
C7—C6—C11—C10	−1.9 (9)	C23—C24—C25—C26	2.9 (10)
C5—C6—C11—C10	171.2 (5)	Br3—C24—C25—C26	−178.4 (5)
C9—C10—C11—C6	−0.9 (10)	C22—C21—C26—C25	2.7 (10)
N2—C5—C12—C13	−137.7 (6)	C20—C21—C26—C25	−175.0 (6)
C6—C5—C12—C13	95.7 (7)	C24—C25—C26—C21	−2.8 (10)
N2—C5—C12—C17	45.9 (8)		

### Hydrogen-bond geometry (Å, °)

**Table:**

*D*—H···*A*	*D*—H	H···*A*	*D*···*A*	*D*—H···*A*
C20—H20A···O^i^	0.93	2.60	3.480 (7)	159
C2—H2A···O	0.97	2.28	2.710 (7)	106
C20—H20A···O	0.93	2.49	2.821 (8)	101

Symmetry codes: (i) −*x*+1, −*y*+2, −*z*+1.

## References

[bb1] Cremer, D. & Pople, J. A. (1975). *J. Am. Chem. Soc.* **97**, 1354–1358.

[bb2] Enraf–Nonius (1994). *CAD-4 EXPRESS* Enraf–Nonius, Delft, The Netherlands.

[bb3] Harms, K. & Wocadlo, S. (1995). *XCAD4* University of Marburg, Germany.

[bb4] North, A. C. T., Phillips, D. C. & Mathews, F. S. (1968). *Acta Cryst.* A**24**, 351–359.

[bb5] Qian, Y., Zhang, H.-J., Zhang, H., Xu, J. & Zhu, H.-L. (2010). *Bioorg. Med. Chem.* **18**, 4991–4996. 10.1016/j.bmc.2010.06.003 20594859

[bb6] Sheldrick, G. M. (2008). *Acta Cryst.* A**64**, 112–122. 10.1107/S0108767307043930 18156677

[bb7] Shi, Y., Chen, Q.-X., Wang, Q., Song, K.-K. & Qiu, L. (2005). *Food Chem.* **92**, 707–712.

[bb8] Teng, Y.-B., Dai, Z.-H. & Wu, B. (2011). *Acta Cryst.* E**67**, o697. 10.1107/S1600536811006210 PMC3051918 21522442

[bb9] Wu, B., Zhou, L. & Cai, H.-H. (2008). *Chin. Chem. Lett.* **19**, 1163–1166.

